# Micro-Computed Tomography Detection of Gold Nanoparticle-Labelled Mesenchymal Stem Cells in the Rat Subretinal Layer

**DOI:** 10.3390/ijms18020345

**Published:** 2017-02-08

**Authors:** Pooi Ling Mok, Sue Ngein Leow, Avin Ee-Hwan Koh, Hairul Harun Mohd Nizam, Suet Lee Shirley Ding, Chi Luu, Raduan Ruhaslizan, Hon Seng Wong, Wan Haslina Wan Abdul Halim, Min Hwei Ng, Ruszymah Binti Hj. Idrus, Shiplu Roy Chowdhury, Catherine Mae-Lynn Bastion, Suresh Kumar Subbiah, Akon Higuchi, Abdullah A. Alarfaj, Kong Yong Then

**Affiliations:** 1 Department of Biomedical Science, Faculty of Medicine and Health Sciences, Universiti Putra Malaysia, 43400 UPM Serdang, Selangor, Malaysia; avin.keh@gmail.com (A.E.-H.K.); suetlee.ding@gmail.com (S.L.S.D.); 2 Genetics and Regenerative Medicine Research Center, Universiti Putra Malaysia, 43400 UPM Serdang, Selangor, Malaysia; sureshkudsc@gmail.com; 3 Department of Ophthalmology, Hospital Sultanah Aminah, 80100 Johor Bahru, Johor, Malaysia; sue_ngein@yahoo.com; 4 Department of Ophthalmology, Faculty of Medicine, UKM Medical Center, 56000 Cheras, Kuala Lumpur, Malaysia; hairulnizam@ppukm.ukm.edu.my (H.H.M.N.); ruhaslizan89@gmail.com (R.R.); whs1975@gmail.com (H.S.W.); afifiyad@yahoo.co.uk (W.H.W.A.H.); maelynnbdr@gmail.com (C.M.-L.B.); 5 Centre for Eye Research Australia, Royal Victorian Eye & Ear Hospital, Melbourne 3002, Australia; cluu@unimelb.edu.au; 6 Department of Surgery (Ophthalmology), The University of Melbourne, Melbourne 3002, Australia; 7 Tissue Engineering Centre, Universiti Kebangsaan Malaysia Medical Center, 56000 Cheras, Kuala Lumpur, Malaysia; angelaster3@gmail.com, (M.H.N.); shipluchy_56@yahoo.com (S.R.C.); 8 Department of Physiology, Universiti Kebangsaan Malaysia Medical Center, 56000 Cheras, Kuala Lumpur, Malaysia; ruszyidrus@gmail.com; 9 Department of Medical Microbiology and Parasitology, Universiti Putra Malaysia, 43400 UPM Serdang, Selangor, Malaysia; 10 Department of Chemical and Materials Engineering, National Central University, Jhong-li, Taoyuan 32001, Taiwan; akon.higuchi@gmail.com; 11 Department of Reproduction, National Research Institute for Child Health and Development, Tokyo 157-8535, Japan; 12 Department of Botany and Microbiology, King Saud University, Riyadh 11451, Saudi Arabia; aalarfajj@ksu.edu.sa

**Keywords:** mesenchymal stem cells, gold nanoparticles, stem cell tracking, rat subretinal layer, transmission electron microscopy, micro-computed tomography

## Abstract

Mesenchymal stem cells are widely used in many pre-clinical and clinical settings. Despite advances in molecular technology; the migration and homing activities of these cells in in vivo systems are not well understood. Labelling mesenchymal stem cells with gold nanoparticles has no cytotoxic effect and may offer suitable indications for stem cell tracking. Here, we report a simple protocol to label mesenchymal stem cells using 80 nm gold nanoparticles. Once the cells and particles were incubated together for 24 h, the labelled products were injected into the rat subretinal layer. Micro-computed tomography was then conducted on the 15th and 30th day post-injection to track the movement of these cells, as visualized by an area of hyperdensity from the coronal section images of the rat head. In addition, we confirmed the cellular uptake of the gold nanoparticles by the mesenchymal stem cells using transmission electron microscopy. As opposed to other methods, the current protocol provides a simple, less labour-intensive and more efficient labelling mechanism for real-time cell tracking. Finally, we discuss the potential manipulations of gold nanoparticles in stem cells for cell replacement and cancer therapy in ocular disorders or diseases.

## 1. Introduction

Regenerative medicine is a fast-growing medical field that encompasses a wide array of techniques to replace and repair damaged tissues within the human body. Mesenchymal stem cells (MSCs), a type of adult stem cell, are widely studied owing to their potential in cellular-based regenerative therapies for treating a variety of medical conditions, including autoimmune [1], cardiovascular [2], and neurodegenerative [3] diseases. These stem cells are also utilized in the repair of bones and cartilage [4]. More recently, there has been growing interest in the use of MSCs to treat retinal diseases [5], partly due to their ability to differentiate into neurons [6]. In addition to being able to differentiate into different cell types [7], MSCs exert reparative effects by migrating to damaged tissues [8,9]. This cellular migration can be monitored for various scientific purposes with the use of histological techniques; however, the process is invasive, time-consuming, and does not allow for real-time tracking [10]. Thus, a more suitable method is required for investigating MSCs in vivo.

Nanoparticles are widely used to facilitate the study of the biodistribution, homing, and cell fate of transplanted cells in living animal models [11]. Gold nanoparticles (GNPs) are one such example, which can be modified to possess different sizes, shapes, and conjugated molecules that ultimately change the physical characteristics of the particles to suit different research needs [12,13]. The GNPs have been demonstrated to not affect the proliferation or cellular functions of MSCs, making them a viable option for in vivo cell tracking [14,15,16]. Moreover, no acute cellular toxicity was associated with the use of GNPs in human cells [17]. To date, GNPs have been widely used in a variety of research applications with MSCs, including as a contrast agent for cell tracking [18] as well as in differentiation studies [19,20] and anti-cancer research via photothermal therapy with cell-based vectors [21]. Thus, GNPs are attractive tools in MSC research. As such, we here report a simple protocol for the labelling of human Wharton’s jelly-derived MSCs (hWJ-MSCs) with GNPs, as well as their detection following subretinal injection in the Royal College of Surgeon (RCS) rat model based on micro-computed tomography (micro-CT) and verification by electron microscopy.

## 2. Results

### 2.1. Characteristics of MSCs

**Figure 1:**
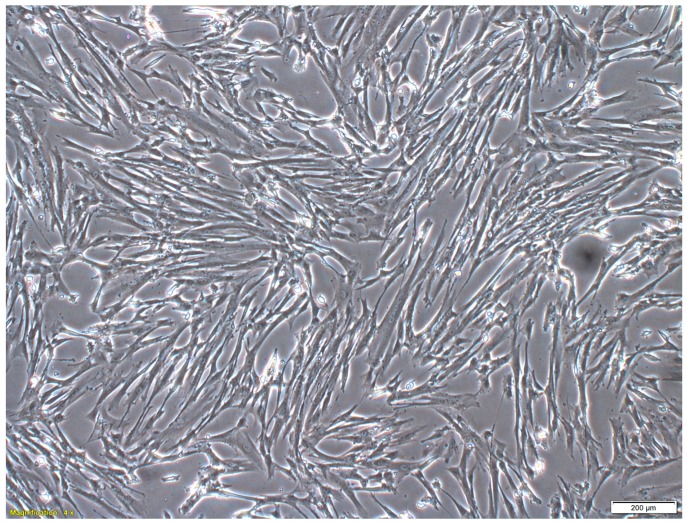
Morphology of human Wharton’s jelly-derived mesenchymal stem cells (hWJ-MSCs). The culture-expanded cells (passage 3) were viewed under a phase-contrast microscope at a total magnification of 40×. The cells showed a fibroblast-like phenotype. The scale bar denotes 200 µm.

**Figure 2:**
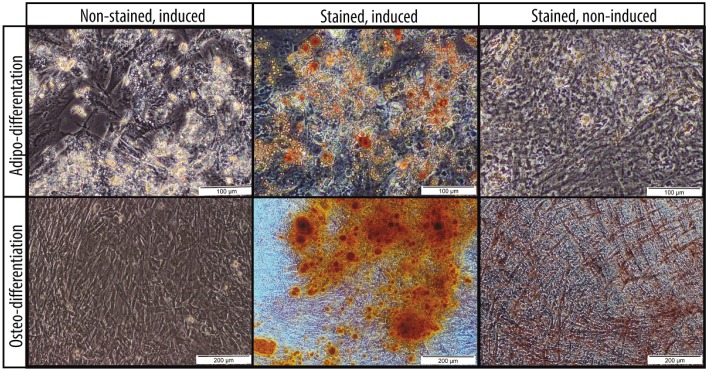
Adipo- and osteo-differentiation of human Wharton’s jelly-derived mesenchymal stem cells (hWJ-MSCs). The presence of adipocytes was visualized by Oil Red O staining after the cells were induced for adipogenic differentiation as compared to the non-induced hWJ-MSCs (200× total magnification). The scale bars denote 100 µm. The presence of osteocytes was visualized by Alizarin Red S staining after the cells were induced for osteogenic differentiation as compared to the non-induced hWJ-MSCs (100× total magnification). The scale bars denote 200 µm.

**Figure 3:**
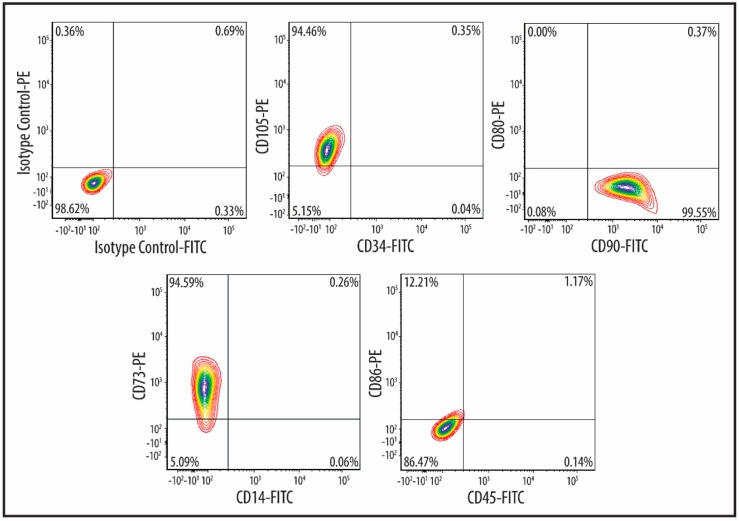
Immunophenotypic analysis of human Wharton’s jelly-derived mesenchymal stem cells (hWJ-MSCs) using the FACSAria III system. The cells showed positive expression of CD105, CD90, and CD73, but not of CD34, CD45, CD14, CD80, and CD86.

The hWJ-MSCs exhibited a typical fibroblast-like morphology (Figure 1), and could differentiate into cells of the adipogenic and osteogenic lineages, which were visualized by Oil Red O and Alizarin Red S staining, respectively (Figure 2). Apart from this multipotent feature, flow cytometric analysis of the culture-expanded cells displayed a clear immunophenotypic characteristic (Figure 3). Specifically, the hWJ-MSCs expressed CD73 (94.6%), CD90 (100%), and CD105 (99.6%), but did not express CD14 (0.1%), CD34 (0%), CD45 (0.1%), CD80 (0%), and CD86 (12.2%). Taken together, these results confirmed that the cells were indeed MSCs, and the culture was not contaminated by any hematopoietic cells.

### 2.2. Quality of GNPs

**Figure 4:**
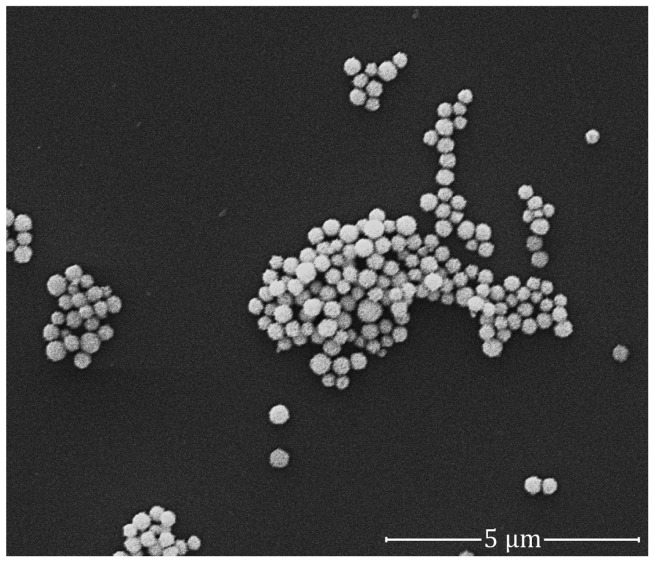
An image of the colloidal GNPs produced by a scanning electron microscope at 20,000× magnification. The particles showed spherical shapes of approximately 80 nm in diameter. The scale bar denotes 5 µm.

The colloidal GNPs were observed under a scanning electron microscope (SEM) to determine the size and shape of the particles. The colloid consisted of homogenous spherical nanoparticles with a size of approximately 80 nm (Figure 4). This step ensured that the particles were in good functioning condition for the subsequent experiment. The absorbance measurement of the particles had a single narrow absorbance peak at 545 nm [5].

### 2.3. Cellular Uptake of GNPs by MSCs

**Figure 5:**
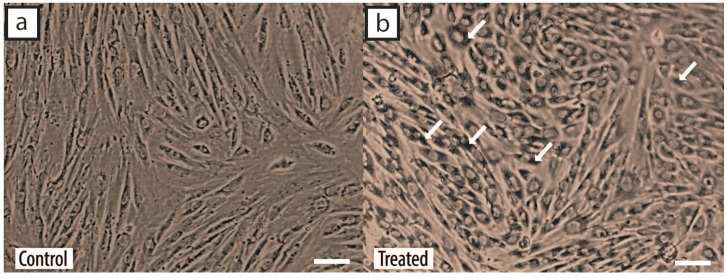
Cellular uptake of gold nanoparticles (GNPs). (**a**) Phase-contrast microscopic image of unlabelled human Wharton’s jelly-derived mesenchymal stem cells (hWJ-MSCs). The scale bar denotes 100 µm; (**b**) phase-contrast microscopic image of GNP-labelled hWJ-MSCs, following 24 h of incubation with 1.4 × 10^8^ GNPs/mL. The white arrows mark the presence of GNPs in the cytoplasm. The scale bar denotes 100 µm.

**Figure 6:**
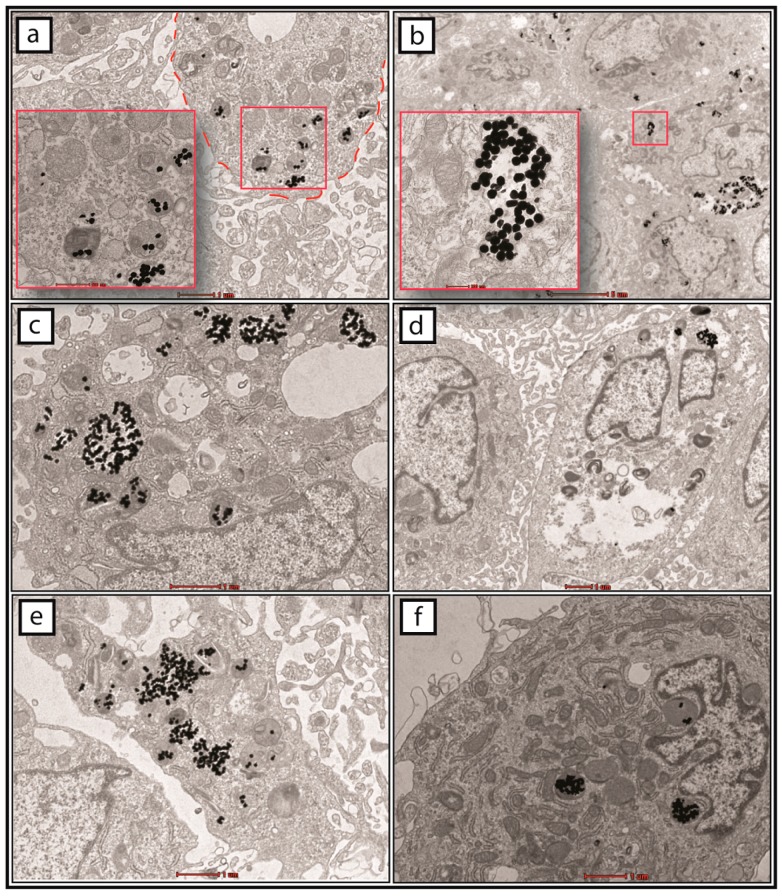
Imaging of gold nanoparticles (GNPs) in the human Wharton’s jelly-derived mesenchymal stem cells (hWJ-MSCs) by transmission electron microscopy (TEM). The treated cells were harvested, pelleted, and processed after incubation with GNPs for TEM observations. (**a**–**f**) Spherical black particles were found to be internalized in small vesicles such as the endosomes. The red dash lines in (**a**) demarcates the boundary of a single cell. The scale bars denote 1 µm (**a**,**c**–**f**) and 5 µm (**b**). The scale bars denote 500 and 200 nm in inset images of (**a**,**b**), respectively.

The cells were incubated with 1.4 × 10^8^ GNPs/mL in a six-well plate for 24 h. The supernatants were discarded and the cells washed with sterile phosphate-buffered saline (PBS) before phase-contrast microscopic images of the GNP-labelled cells were taken. As shown in Figure 5b, the presence of black particles was detected in the cytoplasm of the GNP-labelled hWJ-MSCs, whereas no black particles could be observed in the control cells (Figure 5a) cultured with sterile deionized water only. Before injection into the subretinal layer of the rats, the cells were resuspended in sterile Hanks’ balanced salt solution (HBSS). The pelleted cells were also assessed for the uptake of GNPs using transmission electron microscopy (TEM). As shown in Figure 6, a large number of enveloped (small vesicles, endosomes, etc.) black particles were detected within the cytoplasm of the MSCs.

### 2.4. Tracking of GNP-Labelled MSCs by Micro-CT

**Figure 7:**
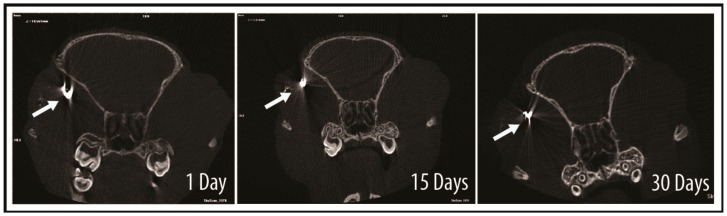
In vivo tracking of cells labelled with gold nanoparticles (GNPs) using micro-computed tomography (micro-CT). Micro-CT scanning showed hyperdensity in the superotemporal section 24 h after injection, as indicated by the white arrow. The hyperdensity remained detectable even after days 15 and 30.

**Figure 8:**
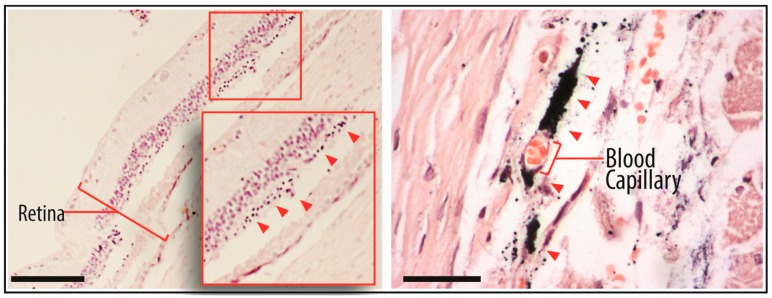
The eyes were harvested and processed for TEM imaging. The sections were stained with Toluidine Blue to localize the target tissue viewing area. (**Left**) Black particles were present in the subretinal layer, as observed under a bright-field imaging microscopy system at a total magnification of 40×, and indicated by the red arrows; (**right**) some particles were also found near the choroidal capillary of a tissue section at a total magnification of 400×.

**Figure 9:**
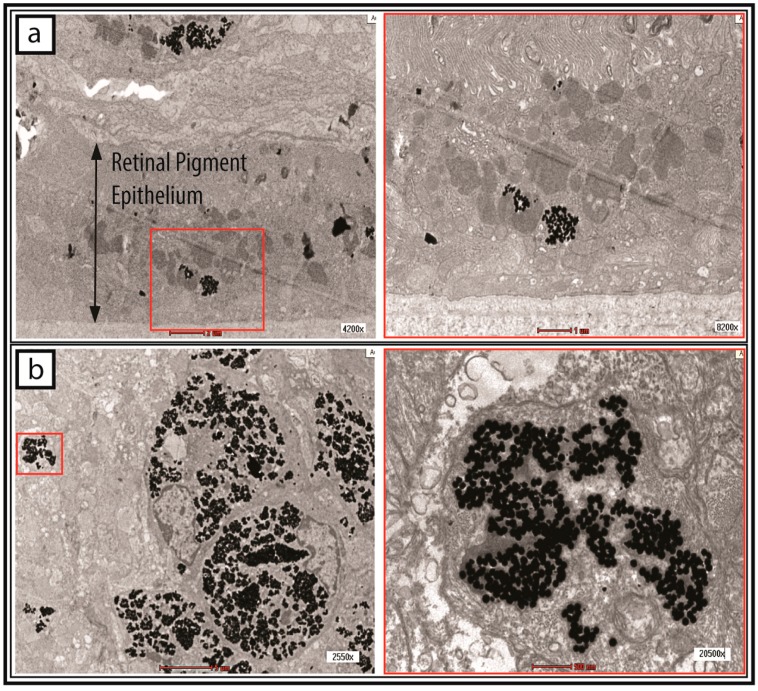
Detection of gold nanoparticle (GNP)-labelled cells in the subretinal layer of the eye by transmission electron microscopy (TEM). (**a**) GNP-labelled cells were found to be present in the retinal pigmented epithelium; the scale bar denotes 2 µm (**left**) and 1 µm (**right**); (**b**) The cells containing the black particles incorporated themselves within the host tissue; the scale bar denotes 5 µm (**left**) and 500 nm (**right**).

After the cells were incubated with GNPs and injected into the subretinal layer of the rats, they were tracked by micro-CT. The site of injection showed an area of hyperdensity, which remained detectable even at days 15 and 30 (Figure 7). With the aid of bright-field microscopy at the area of interest, the presence of black particles could be observed in the retina and choroidal tissue (Figure 8). After enucleating the eye, the localization of GNP-loaded cells was confirmed in the subretinal layer on day 30 using a transmission electron microscope, and was most notably detected in the retinal pigment epithelium (Figure 9).

## 3. Discussion

In this study, we labelled MSCs with GNPs for the purpose of in vivo cell tracking in RCS rats. The MSCs were successfully culture-expanded from human Wharton’s jelly (hWJ-MSCs) (Figure 1) and were shown to be multipotent, since the cells were able to differentiate into adipocytes and osteocytes, which are both part of the mesoderm lineage (Figure 2). These cells highly expressed CD73, CD90, and CD105, but lacked expression of CD14, CD34, and CD45 (Figure 3), indicating the absence of contaminating hematopoietic cells in the culture [22]. In accordance with previous reports, these cells also demonstrated a lack of the co-stimulatory molecules CD80 and CD86 [23], conferring the ability to escape from immune surveillance. The immunophenotypic characteristics of the cultured cells satisfied the criteria for MSC surface markers as defined by the International Society for Cellular Therapy [24].

MSCs hold immense promise in cellular-based regenerative therapies, but the mechanisms by which these cells repair damaged tissues are complex, and invasive methods are often required for analysis, which may result in the premature death of in vivo models [25]. As such, the ability to track these cells with GNPs in real time before animal sacrifice offers more advantages to researchers, such as observing cellular migration patterns and assessing the effectiveness of cell therapy [26]. Prior to labelling the hWJ-MSCs with GNPs, our assessment on the integrity of the particles showed no signs of dysfunction; the aggregates of GNPs were consistently spherical in shape and were around 80 nm in size (Figure 4). In addition, the particles did not exhibit any shift in absorbance at its optimal wavelength of 545 nm. Following treatment with GNPs, the presence of black spots was observed in the cytoplasm of the cells under a phase-contrast microscope (Figure 5). These spots were the GNPs that were taken up via receptor-mediated endocytosis and retained within the endosomes, which would then fuse with lysosomes for processing before being excreted by the cells [27,28]. We confirmed the presence of the particles by TEM observations of the cells collected following incubation with GNPs (Figure 6a–f). Indeed, the cells contained dark vesicle-bound GNPs that were highly suggestive of endosomes. Evaluation of the supernatants collected from the co-incubation of MSCs and GNPs indicated a null value of absorbance at 545 nm, which demonstrated that all the particles had been taken up by the MSCs.

With the accumulation of GNPs in hWJ-MSCs, the cells could be much more easily tracked and distinguished from the surrounding endogenous tissue in the rat using micro-CT and TEM. The micro-CT image (Figure 7) showed an area of hyperdensity following subretinal post-injection in one eye, and the signal remained detectable even after 30 days. In addition, we observed the presence of GNPs around the choroidal capillary in a tissue section stained by Toluidine Blue (Figure 8). One limitation involving nanoparticle labelling is the non-specific uptake of particles by the mononuclear phagocytic/reticuloendothelial system upon the death of the labelled cells [29]. As is the case for the injection of any cell type into an inflamed tissue site, not all cells can survive in the harsh microenvironment. As such, the injected cells would be ultimately phagocytosed by the surrounding immune cells and transported for clearance via the blood circulation. Indeed, the hepatic clearance of GNPs has been observed [30]. In spite of this potential limitation, the TEM histological images (Figure 9) strongly confirmed the presence of GNP-labelled cells in the subretinal layer of the rat, particularly in the retinal pigment epithelium (RPE). Several studies [31,32,33] have demonstrated that MSCs are capable of differentiating into the RPE. Therefore, the presence of GNPs in the RPE could indicate that the transplanted hWJ-MSCs were incorporated into the host RPE to facilitate cellular repair. It is also possible that the phagocytic nature of the RPE resulted in the uptake of GNPs from phagocytosed hWJ-MSCs, since these cells play a role in the phagocytic maintenance and renewal of photoreceptor outer segments [34,35]. Further studies (e.g., immunohistochemistry) are needed to distinguish between these two possibilities. Overall, the results of this study demonstrated the robustness of our protocol, especially in regards to the long-term presence of GNPs, which were retained 30 days after the transplantation procedure.

Various types of cell trackers have been designed to determine the cell fate in an in vivo system. The choice of cell trackers largely depends on the ease of preparation, cost, and capability of the imaging system, and whether or not it will disturb cellular viability and function. Table 1 describes the cell labelling technologies that are commonly used in pre-clinical research. Several methods have been developed for labelling MSCs. Indirect cell labelling involves the overexpression of reporter genes such as the ferritin (Fth1) [36] and transferrin receptor (TfR1) genes [37] before the cells are imaged by magnetic resonance imaging (MRI). In contrast, MSCs can also be directly labelled with nanoparticles such as GNPs. Other examples include superparamagnetic iron oxide nanoparticles and quantum dots, in which the former is detectable by MRI [38], while the latter is a fluorescence-based technique [39].

Compared to other methods, GNPs are relatively cost-effective and simple to utilize in cell labelling. Only a few reports have been published on the effects of GNP uptake for the cellular viability and function of MSCs. It has been suggested that the size and shapes of nanoparticles could affect the viability and differentiation potential of MSCs. For instance, Fan et al. [40] found that the size of a GNP affected its cellular toxicity and proliferation, i.e., GNPs of smaller sizes caused higher cytotoxicity and reduced the formation of colony-forming unit-fibroblasts by human bone marrow-derived MSCs, and vice versa. Furthermore, Li et al. [16] reported that GNPs could influence osteogenic differentiation by regulating the activation of Yes-associated protein. We have reported the cell viability with LIVE/DEAD staining and proliferation using CellTiter-Glo reagent upon successful labelling with 80 nm of GNPs. Both cell viability and proliferation were not significantly affected by the uptake of GNPs even after 10 days of continuous cell culture [5].

Our current study provides evidence for the successful labelling of hWJ-MSCs with GNPs, and the localization of the labelled cells in the subretinal layer of an RCS rat model. In our previous study [5], we suggested the possible differentiation of unlabelled MSCs into rod photoreceptors, bipolar, and Müller glial cells using the same rat model. Hence, future studies should evaluate the possible effects of GNP for directing the differentiation of MSCs into retinal neuron cells and the underlying mechanisms. This might lead to new knowledge and methods to treat blindness caused by retinal degenerative disorders [41]. Wang et al. [42] reported that a low concentration of gold/Fe_3_O_4_ promoted the differentiation of rat olfactory bulb stem cells into neural precursor cells. Paviolo et al. [43] demonstrated that poly(4-styrenesulfonic acid)-coated and SiO_2_-coated gold nanorods could be taken up by NG108-15 neuronal cells (a neural cell line of neuroblastoma × glioma hybrid). Upon exposure to laser light at the plasmon resonance wavelength, the gold nanorods could induce the differentiation process in nanoparticle-treated cells, as indicated by increases in the neurite length, number of neurites per neuron, and the percentage of neurons with neurites.

The GNP-labelled cells may also offer a new form of treatment for eye cancers such as retinoblastoma and choroidal melanoma, since MSCs possess the ability to migrate to tumour cells [21]. Large GNPs (80 nm) can absorb light and convert it into heat energy [44], leading to cancer cellular destruction. On the other hand, the destruction of MSCs can result in the release of cytokines and trophic factors, which would further aid in tissue repair and regeneration [45]. Specifically, MSCs secrete brain-derived neurotrophic factor, basic fibroblast growth factor, and ciliary neurotrophic factor, which could help in retinal neuron cell regeneration [14]. A clinical trial [46] was initiated in 2011 on the application plasmonic photothermal and GNP technology in stem cells to destroy atherosclerotic plaques. Unfortunately, the trial was terminated due to unknown circumstances. In the case of eye cancer, GNP-loaded cells could be modified at the surface membrane [47,48] to improve the targeting and killing of eye cancer.

## 4. Materials and Methods

### 4.1. MSC Culture Conditions

The culture of MSCs (hWJ-MSCs) was obtained from a local stem cell bank (Cryocord, Cyberjaya, Selangor, Malaysia) and provided in the form of cryovials. The source of the tissue was the umbilical cord-isolated Wharton’s jelly of a woman at full-term gestation, who provided informed consent.

The cell number and viability in the cryovials were determined by performing a Trypan Blue exclusion test. Cells in 1 mL of the cryopreservative were transferred into a 10-mL centrifuge tube containing 9 mL of sterile PBS at room temperature (RT) of 25 °C. The tube was then centrifuged at 200× *g* for 10 min. Following centrifugation, the cells were resuspended in 1 mL of hWJ-MSCs culture medium. The cell concentration and viability were determined using 0.4% Trypan Blue reagent.

The hWJ-MSCs were seeded at a density of 5 × 10^3^ cells/cm^3^ into a T-75 culture flask in 10 mL of culture medium. The culture medium was prepared by supplementing Dulbecco’s modified Eagle’s medium (DMEM)-low glucose with 10% (*v*/*v*) foetal bovine serum (FBS) (Thermo Fisher Scientific, Waltham, MA, USA), 100 U/mL penicillin, 100 µg/mL streptomycin, and 0.25 µg/mL amphotericin b (Thermo Fisher Scientific, Waltham, MA, USA). The culture flasks were immediately stored at 37 °C in an incubator supplemented with 5% CO_2_. The culture medium was changed every three days. The supernatant was aspirated before the addition of 10 mL of fresh culture medium each time, and the cultures were incubated at 37 °C, 5% CO_2_.

Subculture was performed when the cells reached 80%–90% confluence. The supernatant was aspirated before washing and rinsing two times with 5 mL of sterile PBS. The cells were then incubated with 5 mL of 0.25% trypsin/ethylenediaminetetraacetic acid (EDTA; Thermo Fisher Scientific) for 5 min in a CO_2_ incubator at 37 °C. Immediately upon observation of cell detachments, 5 mL of culture medium were added to inactivate the trypsin/EDTA at RT. The medium containing the cells was aspirated using a 10-mL pipette and transferred to a 50-mL centrifuge tube. The tube was then centrifuged at 200× *g* for 10 min. Following centrifugation, the supernatant was discarded and the cell pellet was suspended again in 10 mL of PBS. The tube was then centrifuged at 200× *g* for 10 min. The cell concentration and viability were determined according to the Trypan Blue exclusion test as described above. The above culture protocol was repeated to expand the cells to obtain a sufficient cell number for the experiments. Cell cryopreservation was also carried out to preserve the cells for future studies. For cryopreservation, the cell pellet containing approximately 1 × 10^6^ cells (obtained after centrifugation as indicated above) was suspended in 1 mL of cold cryopreservation medium containing 90% FBS and 10% dimethyl sulfoxide (Thermo Fisher Scientific), and transferred into a 1-mL cryovial. The cryovial was kept overnight at −80 °C in a cryocool box. The cryovial was transferred into a liquid nitrogen tank and stored in the vapour phase until future use.

### 4.2. MSC Characterization

Only cells at passage 3–8 were used for the experiments in this study. To assess the multipotency of the hWJ-MSCs, adipogenic (Cat no. SCR020) and osteogenic (Cat no. SCR028) differentiation assays were performed using kits according to the manufacturer instructions (Merck Millipore, Billerica, MA, USA). To confirm the presence of specific surface markers, the hWJ-MSCs were immunophenotyped by flow cytometry using a cocktail of anti-human CD105 (Cat no. FAB10971P, 5 µL), CD73 (Cat no. 550257, 5 µL), CD90 (Cat no. 555595, 2500 µg), CD34 (Cat no. 348053, 0.625 µg), CD45 (Cat no. 347463, 0.25 µg), CD14 (Cat no. 347493, 0.125 µg), CD80 (Cat no. 340294, 0.15 µg), and CD86 (Cat no. 555658, 5 µL) antibodies (each per 1 × 10^5^ cells). All antibodies, except for CD105 (R&D Systems, Minneapolis, MN, USA), were obtained from BD Pharmingen (BD Biosciences, San Jose, CA, USA).

### 4.3. GNP Labelling: Determination of the Quality of Colloidal GNPs

The colloidal GNPs used in the current study were obtained from BBI (BBI Solutions, Madison, WI, USA). The size of these GNPs is 80 nm with a spherical shape. The sterile colloidal solution was kept under aseptic conditions at 4 °C at all times. To ensure the long-term stability and integrity of the colloidal GNPs, a spectrophotometer was used to determine the optimal absorption wavelength of the colloid.

To achieve this, the bottle containing the colloidal solution was gently inverted five times to homogenously suspend the GNPs. A volume of 1 mL was aspirated using a pipette tip at RT and transferred into cuvettes for spectrophotometry. The absorbance was determined across a wide wavelength range (200–700 nm). The optimal absorption wavelength of the GNPs in deionized water was also determined. One millilitre of the colloid was aspirated into a 1.5-mL Eppendorf tube and centrifuged at 6000× *g* for 10 min to pellet the GNPs. The pelleted GNPs were then added to 1 mL of deionized water and transferred into cuvettes for spectroscopic measurement as described above.

To confirm the physical size and shape of the GNPs, SEM was performed. A volume of 1 mL was aspirated using a pipette tip at RT and directly deposited onto a silicon chip for SEM. The particles were imaged using an FEI Helios Dual-Beam SEM-based measuring system (FEI Company, Hillsboro, OR, USA), which was equipped with a high-performance electron beam column and sample stage.

### 4.4. GNP Labelling: Cell Incubation with GNPs

Prior to the cell labelling experiment with GNPs, the hWJ-MSCs were cultured on a six-well plate at a seeding density of 5 × 10^3^ cells/cm^3^ in 2 mL of culture medium. The cells were incubated in a CO_2_ incubator at 37 °C until reaching 70% confluence (approximately 12–16 h).The supernatant in the six-well plate was discarded and the cells were washed twice using 2 mL of sterile PBS at RT. The cells were then added to 1 mL of DMEM and incubated in a CO_2_ incubator at 37 °C until the addition of GNPs.

The colloidal GNPs in the bottle were inverted five times at RT. One millilitre of the colloid was aspirated into a 1.5-mL Eppendorf tube. The tube was centrifuged at 6000× *g* for 10 min to pellet the GNPs. The supernatant was discarded following centrifugation, and the pelleted GNPs were then resuspended in 1 mL of DMEM to attain a final concentration of 1.4 × 10^8^ particles/mL. The suspended GNPs were then added into each well (200 µL/cm^2^) containing the prepared hWJ-MSCs. The cells were observed under a phase-contrast microscope before stored in a CO_2_ incubator at 37 °C for 24 h.

After 24 h, the culture medium from the wells was harvested and collected in a 15-mL centrifuge tube to determine the remnant GNPs by measuring the absorbance value at 545 nm according to the protocol described in Section 4.3. The cell culture medium was replaced with fresh full culture medium containing 10% (*v*/*v*) FBS, 100 U/mL penicillin, 100 µg/mL streptomycin, and 0.25 µg/mL amphotericin. The six-well plate was then immediately placed in a CO_2_ incubator at 37 °C until subretinal transplantation. For subretinal transplantation, the cells were trypsinized, and washed at least two times before resuspension in HBSS. The cells were immediately used for injection as described below in Section 4.6.

### 4.5. GNP Lableling: Determination of the Cellular Uptake of GNPs

To confirm that all GNPs were taken up by the hWJ-MSCs, the culture medium harvested from the cell incubation step (Section 4.4) was assessed for the presence of GNPs. The collected culture medium was first centrifuged at 6000× *g* for 10 min. The supernatant was then discarded, and 1 mL of deionized water was added. The added deionized water was gently flicked and then transferred into a cuvette for absorbance measurement according to the protocol outlined in Section 4.3.

The presence of GNPs taken up by the cells was visualized with TEM of the cytoplasm. The supernatant was aspirated before washing and rinsing two times with 2 mL of sterile PBS. The cells were incubated with 0.5 mL of 0.25% trypsin/EDTA for 5 min in a CO_2_ incubator at 37 °C. Immediately upon observation of cell detachments, 0.5 mL of the culture medium was added to inactivate the trypsin/EDTA at RT. The medium containing the cells was aspirated using a 1-mL pipette and transferred to a 50-mL centrifuge tube. The tube was then centrifuged at 200× *g* for 10 min. Following centrifugation, the supernatant was discarded and the cell pellet was suspended again in 2 mL of PBS. The tube was then centrifuged at 200× *g* for 10 min. The supernatant was discarded to collect the cell pellet for TEM imaging.

### 4.6. Subretinal Injection of GNP-Labelled MSCs

The study was approved by the Universiti Kebangsaan Malaysia Animal Ethics Committee (UKMAEC) (Approval no: PP/OPHTAL/2011/HASLINA/22-MARCH/363-APRIL-2011-DECEMBER-2012) on March 2011. The animal experiments were conducted according to the guidelines provided by UKMAEC and conformed to the ARVO Statement for the Use of Animals in Ophthalmic and Vision Research. All the instruments used were autoclaved before the cell injection procedure was conducted. The RCS rats used in this experiment were between 18 and 21 days old. The rats were first anesthetized by intraperitoneal injections of ketamine (80 mg/mL) and xylazine (12 mg/mL) (Sigma-Aldrich, St. Louis, MO, USA) based on a 100 µL/10 g body weight dosage. The hind paws were pinched to ensure that the rats were properly anaesthetized, and dilating eye drops were instilled in both eyes. After 5 min, or once adequate anaesthesia was achieved, the rats were positioned under a stereomicroscope on their abdomen with the eyes facing the microscope lens. The eyes and fundus of the rats were checked to ensure that no abnormalities were present. A traction suture was placed on the conjunctiva to expose the site of the intended injection, which was located temporosuperiorly. Conjunctival periotomy was performed to expose the bare sclera. A 30-G sharp needle was then carefully used to introduce a small hole behind the limbus at an oblique angle, and care was taken to not penetrate the needle into the lens. Then, 2 × 10^5^ GNP-labelled MSCs in sterile HBSS solution were loaded into syringes equipped with a 30-G blunt needle.

The blunt needle was then carefully inserted through the same track, and the cells were slowly injected into the subretinal space. After injection, the syringe was carefully retracted, and the site of injection was marked with a nylon suture. The fundus was examined for a subretinal bulge, and eyes with complications such as inadvertent vitreous injection, vitreous haemorrhage, or retinal detachment were excluded. Finally, antibiotic ointment was applied to the eye and the rats were gently placed back in the recs inadvertent viovery chamber until they regained full consciousness.

### 4.7. Micro-CT of Treated Rats

At days 1, 15, and 30 post-injection, the rats injected with GNP-labelled hWJ-MSCs were anesthetized with similar doses described above and carefully fastened onto a CT bed. The bed position was adjusted to fit under the field of view of the scanner. Micro-CT was then conducted by an experienced operator, with a source spot size of 8.88 µm at 45 kVp (with 0.5-mm AI filtering). The rats were finally released from the harness and returned to their cages to be kept under monitoring from time to time.

### 4.8. TEM of Injected MSCs

To track the injected MSCs in the eyes, the rats were sacrificed at 30 days post-injection. The rats were first secured in a restrainer with their tails exposed. Euthanasia was performed using a drug overdose by intravenous injections of sodium pentobarbital (Sigma-Aldrich, St. Louis, MO, USA) into the rat tail vein at a dosage of 150 mg/kg. The skin around the eyes of the rats was stretched to allow the eye to slightly pop out. A pair of dissecting scissors was used to sever the optic nerve, allowing the eyeballs to be harvested. The harvested eye cups containing the injected MSCs were fixed by immersing the samples in sterile 0.1 M phosphate buffer (pH 7.4) with 2.5% glutaraldehyde solution for 1 h at RT. The samples were then washed with 0.2 M phosphate buffer, and the eye cups were treated with 1% osmium tetroxide solution (OsO_4_) in 0.1 M phosphate buffer for 1 h, and then washed at least five times in distilled water.

En bloc staining was performed in 2% aqueous uranyl citrate in the dark for 2 h at 4 °C. The samples were then dehydrated with graded ethanol (35% to 100%, with a 15-min dehydration period each) and treated with epoxy propane before embedding in 812 resin overnight. The embedding moulds were left to cure for 24 h at 60 °C, and then the embedded tissues were sectioned serially using a diamond knife on an ultramicrotome to first achieve a thickness of 500 nm. The sections were stained with 1% Toluidine Blue and viewed under a microscope to identify the site of interest in the retina. Subsequently, 76-nm tissue slices were collected on a slot grid, and then stained with uranyl acetate for 2 h and with lead citrate for 5 min.

Once the sections were washed, TEM images were taken by an experienced operator using a Tecnai G2 Spirit Biotwin system (FEI Company). The uptake of GNPs into the cytoplasm of hWJ-MSCs was assessed and confirmed using TEM following essentially the same protocol as described above. However, the cells were collected by first centrifuging at 400× *g* and washed with PBS twice before cell fixation.

## 5. Conclusions

In conclusion, we have provided a simple protocol for labelling human Wharton’s jelly-derived Mesenchymal Stem Cells (MSCs) with GNPs for in vivo cell tracking. MSCs are suitable candidate for cell replacement therapy, particularly due to their easy isolation and expansion from adult tissues, differentiation potential, homing abilities to site of injury and immunomodulatory properties. Many studies have evinced the potential of MSCs to regenerate retinal neurons and delay visual impairment in retinitis pigmentosa animal models. The current study found that the GNP-labelled cells could be maintained and easily detected for one month post-injection in the subretinal layer using micro-CT. The Royal College of Surgeon rats we used belonged to a transgenic model that has defective retinal pigmented epithelium functions and could ultimately result in retinitis pigmentosa. TEM imaging revealed that the MSCs could have differentiated into retinal neuron cells when exposed to cytokines or trophic factors released by the fast-degenerating photoreceptors. In addition, we also observed possible cell differentiation into the retinal pigmented epithelium in the rats. The coupling of stem cells and nanoparticle technology may offer a relatively new and superior treatment strategy for cell replacement therapy in ocular disorders or diseases. Nanotechnology could also influence cell differentiation through intracellular particle interaction. Future studies should optimize different sizes and shapes to efficiently direct the differentiation of the GNP-loaded cells into retinal neuron cells and test for their toxicity. Lastly, we also suggest the potential use of GNP-loaded stem cells to kill cancer cells such as retinoblastoma in the eyes. Cancer cells are more sensitive to heat released by the particles when exposed to plasmonic photothermal energy. The destruction of MSCs could release important protein factors for simultaneous retinal tissue reconstruction and reparation. This paper supported the notion that nanotechnology may provide alternative research platform in stem cell tracking to new treatment modalities for cancer and regenerative medicine to improve or restore damaged tissues.

## Figures and Tables

**Table 1:** Cell labelling technologies used in preclinical studies.

Cell Labelling Technology	Advantages/Disadvantages	References
Viral or non-viral reporter gene systems	Advantages: Allows monitoring of co-expressed genes.	[49]
	Disadvantages: Labour-intensive and time-consuming in the preparation of transduced cell clones. Radioactive substance is required in a conventional chloramphenicol acetyl transferase reporter system, which is potentially hazardous.	[16,50]
Free organic dyes e.g., PKH26, carboxyfluorescein succinimidyl ester (CFSE)	Advantages: Simple cell-labelling protocol. Long-term cell tracking in both in vitro and in vivo systems, e.g., PKH26.	[51]
	Disadvantages: Possible transfer of dye from labelled to unlabelled cells.	[52]
Organic dye nanoparticles	Advantages: Suitable for living-cell imaging as it demonstrates high fluorescence intensity, large Stokes shift, photostability, and emission in the near-infrared range.	[53]
	Disadvantages: High tendency of organic dye to stick to the cell substrate.	[54]
Superparamagnetic iron oxide nanoparticles (SPIO)	Advantages: Direct tissue targeting of SPIO-labelled cells is feasible with use of an appropriate magnetic field.	[55]
	Disadvantages: Requires cross-linking with a membrane-translocating signal peptide (e.g., HIV-1 Tat protein) or co-incubation with transfection agents to facilitate cellular uptake.	[25]
Semiconductor quantum dots	Advantages: Photostable, possesses size-controlled fluorescence, and the emitted fluorescence has a long lifetime.	[56]
	Disadvantages: High cost of reagents; generation of free radicals may cause cellular toxicity.	[57,58]
Noble metallic nanoparticles e.g., gold nanoparticles	Advantages: Simple cell-labelling protocol and no acute cellular toxicity demonstrated.	[59]
	Disadvantages: Different shapes and sizes could affect stem cell differentiation potential.	[16,40]
